# Activation of the EGFR-PI3K-CaM pathway by PRL-1-overexpressing placenta-derived mesenchymal stem cells ameliorates liver cirrhosis via ER stress-dependent calcium

**DOI:** 10.1186/s13287-021-02616-y

**Published:** 2021-10-24

**Authors:** Se Ho Kim, Jae Yeon Kim, Soo Young Park, Won Tae Jeong, Jin Man Kim, Si Hyun Bae, Gi Jin Kim

**Affiliations:** 1 Department of Biomedical Science, CHA University, Seongnam, 13488 Republic of Korea; 2 Research Institute of Placenta Science, CHA University, Seongnam, 13488 Republic of Korea; 3 Department of Oral Microbiology and Immunology, School of Dentistry and Dental Research Institute, Seoul National University, Seoul, 08826 Republic of Korea; 4 Department of Internal Medicine, Catholic University Medical College, Seoul, 06591 Republic of Korea

**Keywords:** Calcium homeostasis, ER stress, Liver cirrhosis, Liver regeneration, Mitochondria, Placenta-derived mesenchymal stem cells, Phosphatase of regenerating liver-1

## Abstract

**Background:**

Cholesterol accumulation and calcium depletion induce hepatic injury via the endoplasmic reticulum (ER) stress response. ER stress regulates the calcium imbalance between the ER and mitochondria. We previously reported that phosphatase of regenerating liver-1 (PRL-1)-overexpressing placenta-derived mesenchymal stem cells (PD-MSCs^PRL−1^) promoted liver regeneration via mitochondrial dynamics in a cirrhotic rat model. However, the role of PRL-1 in ER stress-dependent calcium is not clear. Therefore, we demonstrated that PD-MSCs^PRL−1^ improved hepatic functions by regulating ER stress and calcium channels in a rat model of bile duct ligation (BDL).

**Methods:**

Liver cirrhosis was induced in Sprague–Dawley (SD) rats using surgically induced BDL for 10 days. PD-MSCs and PD-MSCs^PRL−1^ (2 × 10^6^ cells) were intravenously administered to animals, and their therapeutic effects were analyzed. WB-F344 cells exposed to thapsigargin (TG) were cocultured with PD-MSCs or PD-MSCs^PRL−1^.

**Results:**

ER stress markers, e.g., eukaryotic translation initiation factor 2α (eIF2α), activating transcription factor 4 (ATF4), and C/EBP homologous protein (CHOP), were increased in the nontransplantation group (NTx) compared to the control group. PD-MSCs^PRL−1^ significantly decreased ER stress markers compared to NTx and induced dynamic changes in calcium channel markers, e.g., sarco/endoplasmic reticulum Ca^2+^ -ATPase 2b (SERCA2b), inositol 1,4,5-trisphosphate receptor (IP3R), mitochondrial calcium uniporter (MCU), and voltage-dependent anion channel 1 (VDAC1) (*p < 0.05). Cocultivation of TG-treated WB-F344 cells with PD-MSCs^PRL−1^ decreased cytosolic calmodulin (CaM) expression and cytosolic and mitochondrial Ca^2+^ concentrations. However, the ER Ca^2+^ concentration was increased compared to PD-MSCs (*p  < 0.05). PRL-1 activated phosphatidylinositol-3-kinase (PI3K) signaling via epidermal growth factor receptor (EGFR), which resulted in calcium increase via CaM expression.

**Conclusions:**

These findings suggest that PD-MSCs^PRL−1^ improved hepatic functions via calcium changes and attenuated ER stress in a BDL-injured rat model. Therefore, these results provide useful data for the development of next-generation MSC-based stem cell therapy for regenerative medicine in chronic liver disease.

**Supplementary Information:**

The online version contains supplementary material available at 10.1186/s13287-021-02616-y.

## Introduction

The endoplasmic reticulum (ER) is responsible for various cellular activities, such as protein secretion, synthesis, maturation, translation and folding in eukaryotic cells, and it plays an important role in regulating calcium concentrations [1]. ER stress induces the accumulation of misfolded proteins or calcium depletion in the ER lumen, and these events result in progression to severe stages of several diseases, such as diabetes, obesity, and nonalcoholic fatty liver disease (NAFLD)/nonalcoholic steatohepatitis (NASH) [2]. ER stress in liver fibrosis results in the accumulation of unfolded proteins, very low-density lipoprotein (VLDL), inflammatory cytokines and oxidative stress, especially Ca^2+^ imbalances in the ER lumen [3]. ER stress dysregulates calcium pump channels in chronic liver diseases [4], calcium inflow controls sarco/endoplasmic reticulum Ca^2+^ ATPase (SERCA2b), and calcium release regulates inositol trisphosphate receptor (IP3R), ryanodine receptor 2 (RyR2) and calcium sensor stromal interaction molecule 1 (STIM1) in the ER membrane [5]. SERCA activity is impaired in NAFLD, which leads to imbalanced calcium homeostasis under ER stress [6].

The unfolded protein response (UPR) and abnormal UPR pathway restore normal functioning of the cell and cell death in cases of irreversible disruption. PKR-like ER kinase (PERK) is a key factor of the UPR that induces apoptosis via the transcription factor C/EBP homologous protein (CHOP) and phosphorylation of eukaryotic translation initiation factor 2 alpha (eIF2α) to upregulate the expression of activating transcription factor 4 (ATF4). CHOP-deleted mice showed reduced apoptotic and necrotic hepatocyte death via decreases in the expression of alpha-smooth muscle actin (α-SMA) and transforming growth factor beta-1 (TGF-β1) [7]. However, the role of ER stress-dependent calcium influx in liver diseases, including cirrhosis, is not known.

Ca^2+^-mobilizing growth factors generate Ca^2+^ signals in hepatocytes via receptor tyrosine kinases (RTKs). RTKs are membrane proteins that bind to ligands, such as growth factors, cytokines, and hormones, in the plasma membrane. Several RTKs are expressed in the liver, including hepatocyte growth factor receptor (HGFR), vascular endothelial growth factor receptor (VEGFR), platelet-derived growth factor receptor (PDGF), and epidermal growth factor receptor (EGFR) [8]. EGFR is a key regulator in early inflammation and hepatocyte proliferation in hepatic disease [9]. The activation of EGFR in liver disease affects liver regeneration via the downstream signaling pathway Ras-Raf-MEK-ERK1/2, and it controls the phosphatidylinositol-3-kinase (PI3K)-Akt mechanism [10]. For activation of RTK-based signaling, several ligands bind their specific receptors to activate phospholipase C (PLC) or PI3K, which hydrolyze phosphatidylinositol-4–5-bisphosphate (PIP2) to generate diacylglycerol (DAG) and inositol 1,4,5-trisphosphate (IP3). ER-resident IP3R calcium channels in chronic liver diseases lead to elevated cytosolic and decreased ER calcium levels [11]. A previous report suggested that IGF-1 regulated spliced X-box binding protein 1 (sXBP-1) stability, protein synthesis and Ca^2+^ storage in the ER, which protected beta cell ER stress-mediated cell death at the onset of type 1 and type 2 diabetes [12]. However, the RTKs that regulate Ca^2+^ signaling in cirrhotic rat liver are not known.

Phosphatase of regenerating liver-1 (PRL-1) is a member of a small class of the prenylated PTP family, and it was identified as an immediate early gene in the regenerating rat liver [13]. Increased PRL-1 also regulated the proliferation and migration of mesenchymal stem cells in a liver cirrhosis rat model [14], and it shows high homology between humans and rats/mice [15]. Wang et al. reported that PRL-1 localized to the ER during the mitotic cell cycle [16]. During ER stress, increased PRL-1 suppressed apoptosis and enhanced ER function in a p53-dependent manner in injured cells [17]. A dual specificity tyrosine targeted by PRL-1 in the Ca^2+^ pathway was reported [18]. However, research on PRL-1 direct modulation of the Ca^2+^ pathway is lacking, and the binding of PRL-1 to RTK ligands in the plasma membrane is not known.

Our previous reports demonstrated that placenta-derived MSC (PD-MSC) transplantation enhanced hepatic function via increased antifibrotic effects, proliferation, and autophagy in a chronic liver disease model [19]. PRL-1-overexpressing PD-MSCs (PD-MSCs^PRL−1^) also enhanced hepatic functions by increasing mitochondrial functions and proliferative potential in a chronic liver disease model compared to naïve PD-MSCs [20]. However, the relationship of PD-MSC^PRL−1^ transplantation to the calcium-induced ER stress mechanism in cirrhotic livers is not clear. Therefore, the present study investigated whether PD-MSC^PRL−1^ implantation induced Ca^2+^ influx in injured liver tissues in a rat model of bile duct ligation (BDL), whether it induced Ca^2+^ influx and how it correlated with ER stress. Finally, we evaluated the therapeutic mechanism of PD-MSC^PRL−1^ modulation of ER stress-dependent calcium via RTK signaling in a rat model of BDL.

## Materials and methods

### Cell culture

The institutional review board of CHA Bundang Hospital, Seoul, Republic of Korea, approved the collection and use of samples for research purposes (IRB 07–18). All participants provided written informed consent prior to sample collection. Placentas were extracted from women who were free of medical, obstetrical, and surgical complications and delivered at term (≥ 37 gestational weeks). PD-MSCs were isolated as previously described [21] and cultured in α-modified minimal essential medium (α-MEM; HyClone Logan, UT, USA) supplemented with 10% fetal bovine serum (FBS; Gibco, Carlsbad, CA, USA), 1% penicillin/streptomycin (P/S; Gibco), 1 μg/mL heparin (Sigma-Aldrich, St. Louis, MO, USA), and 25 ng/mL human fibroblast growth factor-4 (hFGF-4; Peprotech, Inc., Rocky Hill, NJ, USA). The PRL-1 plasmid vector was purchased from Origene (#RG200435; Rockville, MD, USA). For overexpression of the PRL-1 gene, naïve PD-MSCs (passage = 7) were transfected using the AMAXA nucleofector system (Lonza, Basel, Switzerland) according to the manufacturer’s instructions as previously described [20]. After transfection for 24 h, the cells were selected using 1.5 mg/mL neomycin. WB-F344 cells (rat liver epithelial cells) were cultured at 37 °C in α-MEM supplemented with 10% FBS (Gibco) and 1% P/S (Gibco). All cells were maintained at 37 °C in a 5% CO_2_ incubator.

### Animal models and transplantation of MSCs

Seven-week-old male Sprague–Dawley rats (Orient Bio, Inc., Seongnam, Republic of Korea) were maintained in an air-conditioned animal facility. Rats were anesthetized via intraperitoneal injection using avertin (2,2,2-tribromoethanol; Sigma-Aldrich) and underwent common BDL to induce liver cirrhosis as previously described. Ten days after BDL induction [22], naïve PD-MSCs (PD-MSCs; *n * = 20) and PD-MSCs^PRL−1^ (PD-MSCs^PRL−1^; *n * = 20) were stained using the PKH67 Fluorescent Cell Linker Kit (Sigma-Aldrich) and injected intravenously through the tail vein (2 × 10^6^ cells/animal) in the transplantation group. Non-transplanted rats (NTx; *n * = 20) and sham control rats (Con; *n * = 5) were maintained. The rats were sacrificed after 1, 2, 3, and 5 weeks to extract liver tissues and blood samples. The Institutional Animal Care Use Committee of CHA University, Seongnam, Republic of Korea, approved the experimental processes and protocols for animal modeling (IACUC-200033).

### Liver histology and serum biochemistry

Liver tissue samples were fixed in 10% neutral buffered formalin (NBF), embedded in paraffin, and sectioned at 5 μm thickness for hematoxylin and eosin (H&E) and Sirius red staining. Representative images of whole sections of the liver were captured and quantified using a digital slide scanner (3DHISTECH, Ltd., Budapest, Hungary). The serum concentrations of alanine aminotransferase (ALT), aspartate aminotransferase (AST), total bilirubin (TBIL), and albumin (ALB) were measured from individual blood samples (Southeast Medi-Chem Institute, Busan, Republic of Korea).

### Immunofluorescence

For analysis of gene expression in liver tissues, samples were sectioned at 6 μm thickness and fixed using 4% paraformaldehyde. Liver tissues from each group were blocked using Protein Block Serum-Free solution (Dako, Santa Clara, CA, USA) for 1 h. The following primary antibody was used: SERCA2b (1:1000; Invitrogen, Carlsbad, CA, USA). The sections were stained with 4′,6-diamidino-2-phenylindole (DAPI; Invitrogen) and observed using confocal microscopy (LSM 700). Representative images were analyzed using ZEN blue software (Zeiss). The experiment was performed at least in duplicate.

### Immunohistochemistry

To determine calcium channel expression in the ER, liver tissues from each group were sectioned at a thickness of 5 μm and fixed with 10% NBF. The fixed tissues were reacted in 3% hydrogen peroxide (H_2_O_2_) in 100% methanol to block endogenous peroxidase activity. The following antibodies were used: anti-CHOP (1:50; Santa Cruz, Dallas, TX, USA), anti-calmodulin (1:100; Novus Biologicals, Littleton, CO, USA) and anti-PCNA (1:500; company). CHOP was performed using Proteinase K (20 μg/mL) (Dako). However, anti-calmodulin and anti-PCNA were used for antigen retrieval before incubation at 4 ℃ overnight. Incubation with horseradish peroxidase-conjugated streptavidin–biotin complex (Dako) and 3,3-diaminobenzidine (EnVision™ Systems, Santa Clara, CA, USA) was performed to generate a chromatic signal. For counterstaining, Mayer’s hematoxylin (Dako) was used. Representative images were captured and quantified using a digital slide scanner (3DHISTECH, Ltd.).

### Quantitative real-time PCR

Total RNA was isolated from samples using TRIzol (Invitrogen). cDNA was synthesized using Superscript III reverse transcription (Invitrogen) according to the manufacturer’s instructions. For determinations of calcium channel factor expression in liver tissues and hepatocytes, qRT-PCR was performed using rat primers (Table 1, designed by BIONEER, Daejeon, Korea) and SYBR Green Master Mix (Roche, Basel, Switzerland) using the CFX Connect™ Real Time System (Bio-Rad, Hercules, CA, USA). Rat GAPDH was used as an internal control for normalization, and each sample was analyzed in duplicate.

**Table 1:** Primer Sequences (Reverse Transcription Polymerase Chain Reaction)

Type	Gene	Sequence (5’–3’)	Tm (℃)	Accession no
Calcium channel	SERCA2b	F: 5’- CCAGTCGATTCTTACAGGTG -3’ R: 5’- GCGATGTTTGTGCCAGAAAA -3’	56	NM_001110823.2
	IP3R	F: 5’- GGAGCCTCTGGTGAAAACGA -3’ R: 5’- CACCCATGTTCCTCAGCAGT -3’	59	NM_001007235.2
	GRP75	F: 5’- TGATGCCAATGGGATTGTGC -3’ R: 5’- CTGCTTCAACACGTTCCTTC -3’	58	NM_001100658.2
	CaM	F: 5’- GTGAGGCATTCCGAGTCTTT -3’ R: 5’- TCATCTGTTAGCTTTTCCCCG -3’	57	NM_031969.3
	STIM1	F: 5’- ACAAGCTTATCAGCGTGGAG -3’ R: 5’- ACTTCCGGAAAGTCTCCTCA -3’	57	NM_001108496.2
	VDAC1	F: 5’- AACAGTAACACTCGCTTTGG -3’ R: 5’- TTGACGTTCTTGCCATCCAG -3’	57	NM_031353.2
	MCU	F: 5’- GGGGTGTTTCTCCGACAACT -3’ R: 5’- GGAGGTCTCTCTTTGGTGGC -3’	59	NM_001106398.1
Internal control	GAPDH	F: 5’- TCCCTCAAGATTGTCAGCAA -3’ R: 5’- AGATCCACAACGGATACATT -3’	58	NM_017008.4

### Western blotting

Homogenized liver tissues and samples were lysed in RIPA buffer containing a protease inhibitor cocktail (Roche) and phosphatase inhibitor (Sigma-Aldrich). Quantified protein extracts (40 μg) were loaded in 6 ~ 15% sodium dodecyl sulfate polyacrylamide gel electrophoresis (SDS-PAGE). The separated proteins were transferred to polyvinylidene difluoride (PVDF) membranes (Bio-Rad). The following primary antibodies were used: anti-SERCA2b (1:500; Invitrogen), anti-mitochondrial calcium uniporter (MCU) (1:1000; Invitrogen), anti-IP3R (1:800; Cell Signaling Technology), anti-voltage-dependent anion-sensitive channel 1 (VDAC1), anti-total-eukaryotic initiation factor 2α (eIF2α), anti-phospho-eIF2α, anti-activating transcription factor 4 (ATF4), anti-CHOP, anti-PI3K-p110α (1:1000; all from Cell Signaling Technology), anti-PI3K-p85 (1:3000; BD Biosciences, San Jose, CA, USA), anti-PERK (1:200; Santa Cruz), anti-GRP75 (1:500; Abcam), and anti-calmodulin (1:500; Novus Biologicals). The loading control was anti-GAPDH (1:2000; AbFrontier, Seoul, Republic of Korea). After the membranes were washed, the following secondary antibodies were used: anti-mouse IgG and anti-rabbit IgG (1:8,000; all from Bio-Rad). The protein bands were detected using a Clarity Western ECL kit (Bio-Rad) and a ChemiDoc imaging system (Bio-Rad).

### Calcium influx using a fluorescence resonance energy transfer (FRET) biosensor

For confirmation of calcium influx in hepatocytes, cytoplasm-targeting CMV-Y-GECO1, ER-targeting CMV-ER-LAR-GECO1, and mitochondria-targeting CMV-mito-GEM-GECO1 were purchased (Addgene, Watertown, MA, USA). Cells were transfected using Lipofectamine 2000 (Thermo Fisher Scientific) for 4 h and treated with TG (500 nM) for 24 h. For simultaneous monitoring of the Ca^2+^ concentration in the ER, cytoplasm, and mitochondria, widefield imaging was performed using a confocal microscope (LSM880; Carl Zeiss) at 37 °C in a humidified atmosphere with 5% CO_2_.

### Phospho-RTK proteome profiler array

For analysis of the interaction of PRL-1 and RTKs, a human phospho-RTK Array kit (R&D Systems, Minneapolis, USA) was used following the manufacturer’s assay procedures. Briefly, cell lysates (1 × 10^7^ cells) were diluted and incubated overnight with an anti-phospho-tyrosine-HRP detection antibody. Captured signals represented the amount of phosphorylated protein.

#### Statistical analysis

All experiments were performed in duplicate or triplicate. The results are presented as the means ± standard deviation (SD). Student’s *t* test was performed for groupwise comparisons, and a *p*-value less than 0.05 was considered statistically significant. One-way ANOVA was performed for pairwise comparisons and multiple paired t tests.

## Results

### PD-MSCs^PRL−1^ decreased ER stress in a rat model of BDL and hepatocytes

**Fig. 1:**
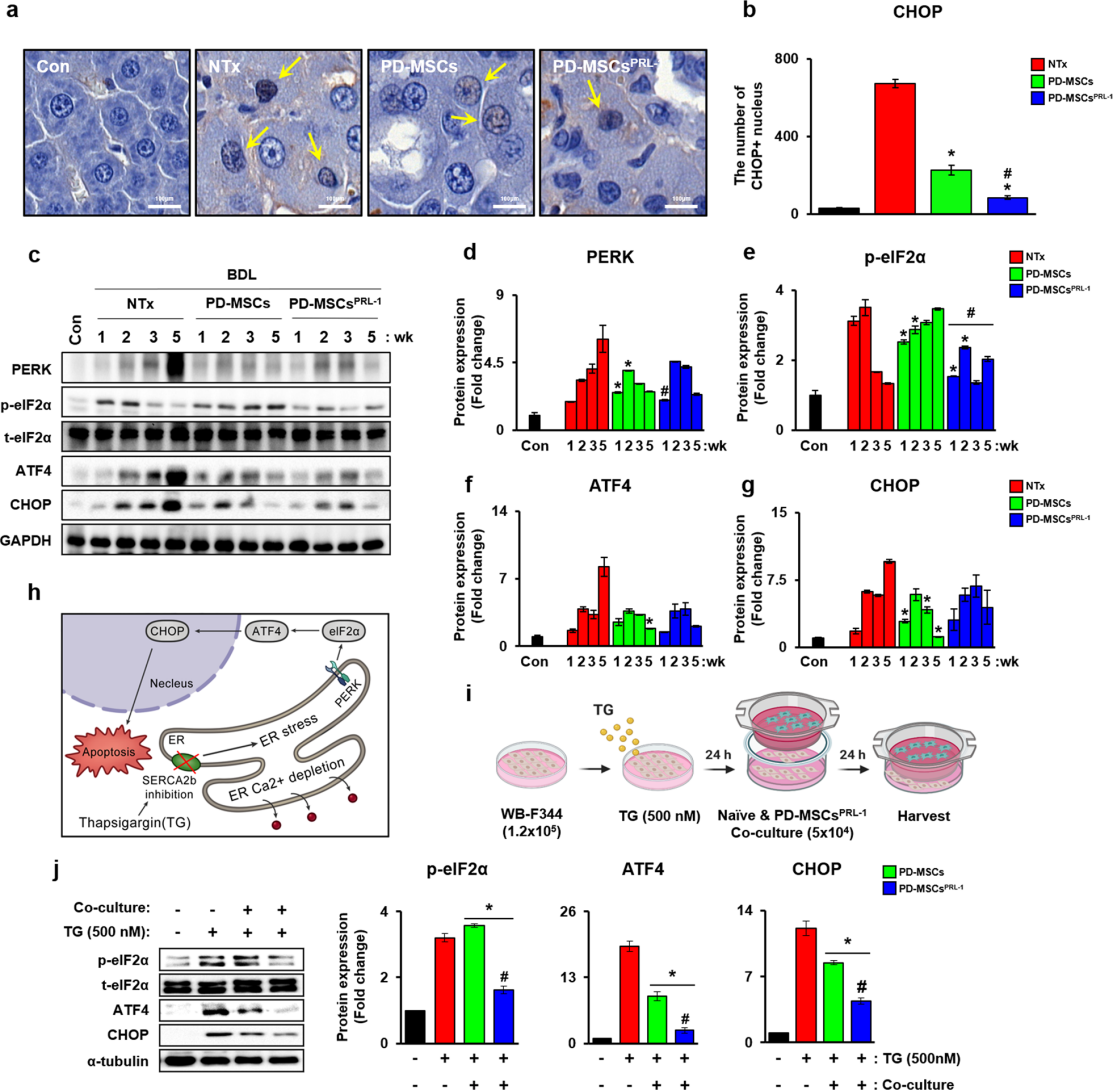
PD-MSC^PRL−1^ transplantation decreased ER stress in a BDL rat model and hepatocytes. **a** Representative IF images of CHOP in cirrhotic livers at 5 weeks using IHC. **b** The number of CHOP-positive cells translocated into the nucleus in a cirrhotic liver. **c** Western blotting of ER stress markers (e.g., PERK, eIF2α, ATF4, and CHOP) in a rat BDL. **d-g** Quantification of ER stress markers (e.g., PERK, total and phosphorylated eIF2 alpha; p-eIF2α, ATF4, and CHOP) following PD-MSC^PRL−1^ transplantation in pooled BDL-injured rat liver protein (*n* = 5/group) at 1, 2, 3, and 5 weeks. **h** A schematic diagram showing TG-induced ER stress. **i** A schematic diagram describing TG (500 nM)-treated WB-F344 cells cocultured with PD-MSCs or PD-MSC^PRL−1^ for 24 h. **j** Western blotting and the intensities of ER stress markers in WB-F344 cells. α-tubulin was used as a loading control. Data from each group are shown as the means ± SD and were assessed using Student’s t test. *p  < 0.05 *vs.* NTx, #*p* < 0.05 *vs.* PD-MSCs. ATF4, activating transcription factor 4; BDL, bile duct ligation; CHOP, C/EBP homologous protein; p-eIF2α, phosphorylated eIF2 alpha; PRL-1, phosphatase of regenerating liver-1; TG, thapsigargin

PERK-eIF2α-ATF4-CHOP signaling in the UPR pathway is implicated in liver diseases [23]. To analyze UPR pathway activation by PD-MSC^PRL−1^ transplantation, we assessed the expression of CHOP, which is an ER stress-mediated transcription factor, in liver tissues using immunohistochemistry (IHC) (Fig. 1a). The expression of CHOP in the nuclei of hepatocytes was significantly increased in the BDL-induced NTx group compared to the normal control (Con) group. Notably, the CHOP level was reduced in PD-MSCs^PRL−1^, and CHOP translocation into the nucleus was significantly decreased (Fig. 1b, *p  < 0.05).

Phosphorylated eIF2α (p-eIF2α) expression was increased at 1 and 2 weeks in the NTx group, but the expression of PERK, ATF4 and CHOP decreased (Fig. 1c). In contrast, the transplantation groups (Tx) showed decreased levels compared to the NTx group. Notably, the expression of PERK and p-eIF2α was significantly reduced in the PD-MSC^PRL−1^ group (Fig. 1d–g, *p  < 0.05). Thapsigargin (TG) is an inhibitor of SERCA2b in hepatocyte-specific calcium channels on the ER membrane, and it resulted in cellular apoptosis (Fig. 1h). To induce ER stress in hepatocytes, we analyzed the expression of ER stress markers after 500 nM TG treatment for 24 h regardless of PD-MSC and PD-MSC^PRL−1^ cocultivation (Fig. 1i). The protein levels of ER stress markers in WB-F344 cells treated with TG were substantially increased. However, their expression levels were significantly decreased after PD-MSC cocultivation. PD-MSC^PRL−1^ cocultivation resulted in significantly reduced expression compared to naïve PD-MSCs (Fig. 1j, *p  < 0.05). These findings indicated that PD-MSCs^PRL−1^ more efficiently decreased ER stress than naïve PD-MSCs in cirrhotic rat livers and hepatocytes.

### PD-MSCs^PRL−1^ regulated calcium channels in a rat model of BDL and hepatocytes

**Fig. 2:**
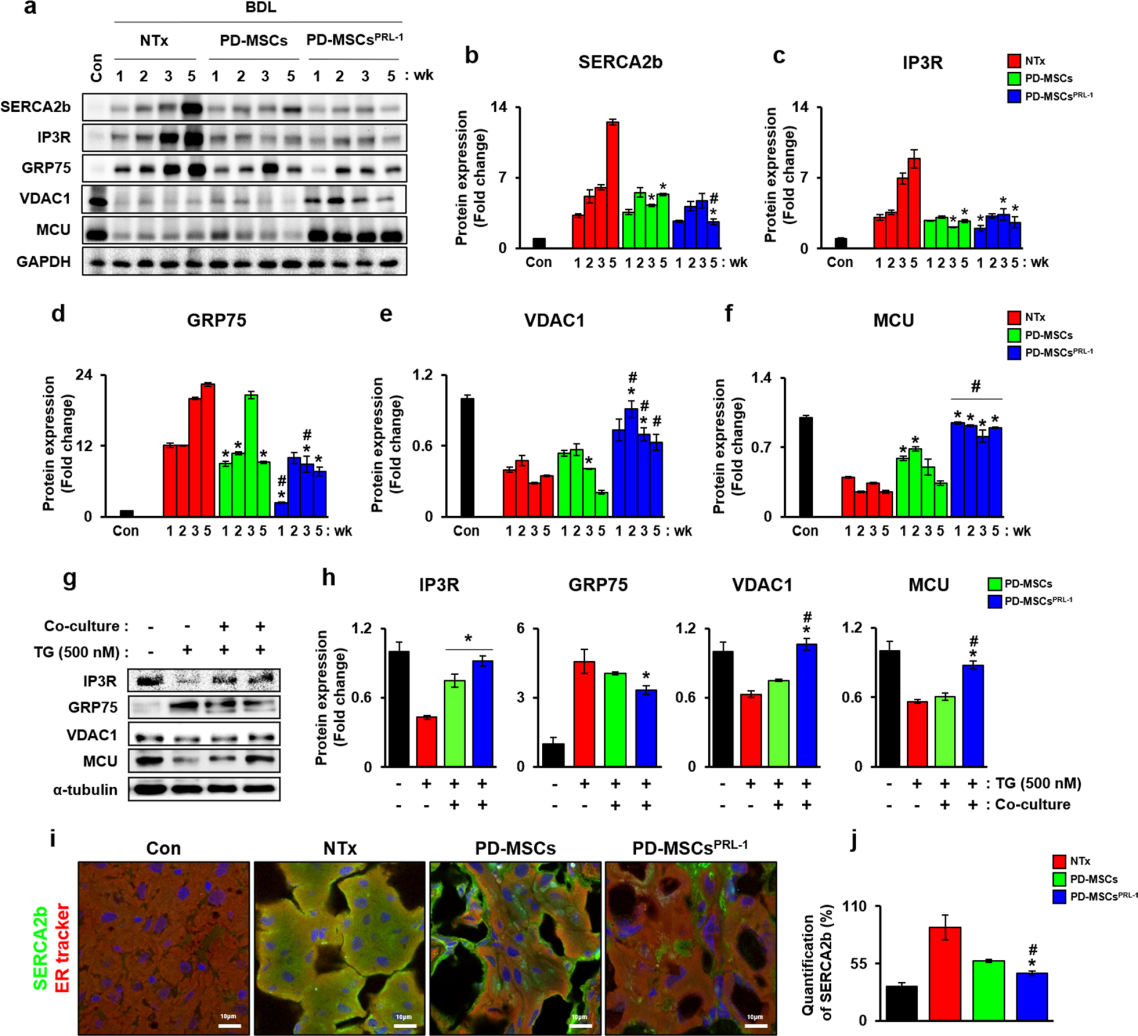
PD-MSC^PRL−1^ transplantation regulated calcium channels in a rat model of BDL and hepatocytes. **a** Western blotting and **b-f** the intensities of calcium channels (e.g., SERCA2b, IP3R, glucose regulated protein 75; GRP75, voltage-dependent anion channel 1; VDAC1, and mitochondria calcium uniporter; MCU) following PD-MSC^PRL−1^ transplantation in BDL-induced rat liver protein measured using glycraldehyde-3-phosphate dehydrogenase (GAPDH) as a loading control. **g** Western blotting and **h** the intensities of calcium channels (e.g., IP3R, GRP75, VDAC1, MCU, and CaM) in TG (500 nM)-treated WB-F344 cells cocultured with PD-MSCs or PD-MSCs^PRL−1^ for 24 h. α-Tubulin was used as a loading control. **i** Representative images and **j** quantification of SERCA2b (green) and ER Tracker (red) in BDL-injured rat livers at 5 weeks using IF staining. DAPI (blue) was used for counterstaining. Data from each group are shown as the means ± SD and were assessed using Student’s t test. *p  < 0.05 *vs.* NTx, #*p * < 0.05 *vs. *PD-MSCs. BDL, bile duct ligation; CaM, calmodulin; GAPDH, glycraldehyde-3-phosphate dehydrogenase; GRP75, glucose regulated protein 75; IP3R, inositol trisphosphate receptor; MCU, mitochondrial calcium uniporter; NTx, nontransplantation; PRL-1, phosphatase of regenerating liver-1; SERCA2b, sarco/endoplasmic reticulum Ca^2+^ ATPase; VDAC1, voltage-dependent anion channel 1

A calcium imbalance between the ER and mitochondria in the liver leads to the development of chronic metabolic diseases and impaired organelle function [24]. Therefore, we confirmed the expression levels of calcium channels in the ER membrane (e.g., SERCA2b and IP3R) and mitochondria (e.g., VDAC1 and MCU) and ER-mitochondrial Ca^2+^ transfer factors (e.g., GRP75) in a rat model of BDL using Western blotting (Fig. 2a). The expression levels of SERCA2b, IP3R, and GRP75 were increased in the NTx group compared to the normal group. (Fig. 2b–d, *p  < 0.05). In contrast, all Tx groups showed decreased levels compared to the NTx group. GRP75 levels were significantly decreased in the PD-MSC^PRL−1^ group at 1 and 3 weeks. However, IP3R and GRP75 expression in the PD-MSC^PRL−1^ group was dramatically increased compared to the PD-MSC group (Additional file 1: Figs. S2a and b). Notably, the mRNA and protein levels of VDAC1 and MCU were increased in the PD-MSC^PRL−1^ Tx group (Fig. 2e and f, Additional file 1: Fig. S2c and d, *p  < 0.05). TG treatment induced a decrease in the expression levels of IP3R, VDAC1, and MCU, and the expression levels of GRP75 and CaM were increased in the WB-F344 hepatocyte cell line. PD-MSC^PRL−1^ cocultivation significantly altered the expression levels of IP3R, VDAC1, MCU, GRP75, and CaM compared to naïve PD-MSCs, but no significant change was found in the mRNA levels between the treated group and the cocultivation group (Additional file 1: Fig. S2e–i, *p  < 0.05). However, protein levels were decreased (Fig. 2g and h, *p  < 0.05). We also confirmed the degree of colocalization with SERCA2b and ER Tracker using immunofluorescence (IF) staining (Fig. 2i). The expression of SERCA2b in the NTx group was increased, but the expression was significantly decreased in the PD-MSC^PRL−1^ group compared to the NTx group and the PD-MSC group (Fig. 2j, *p  < 0.05). These results indicated that PD-MSCs^PRL−1^ efficiently regulated calcium channels between the ER and mitochondria in cirrhotic rat livers and hepatocytes.

### PD-MSCs^PRL−1^ regulated calcium influx in rat hepatocytes

**Fig. 3:**
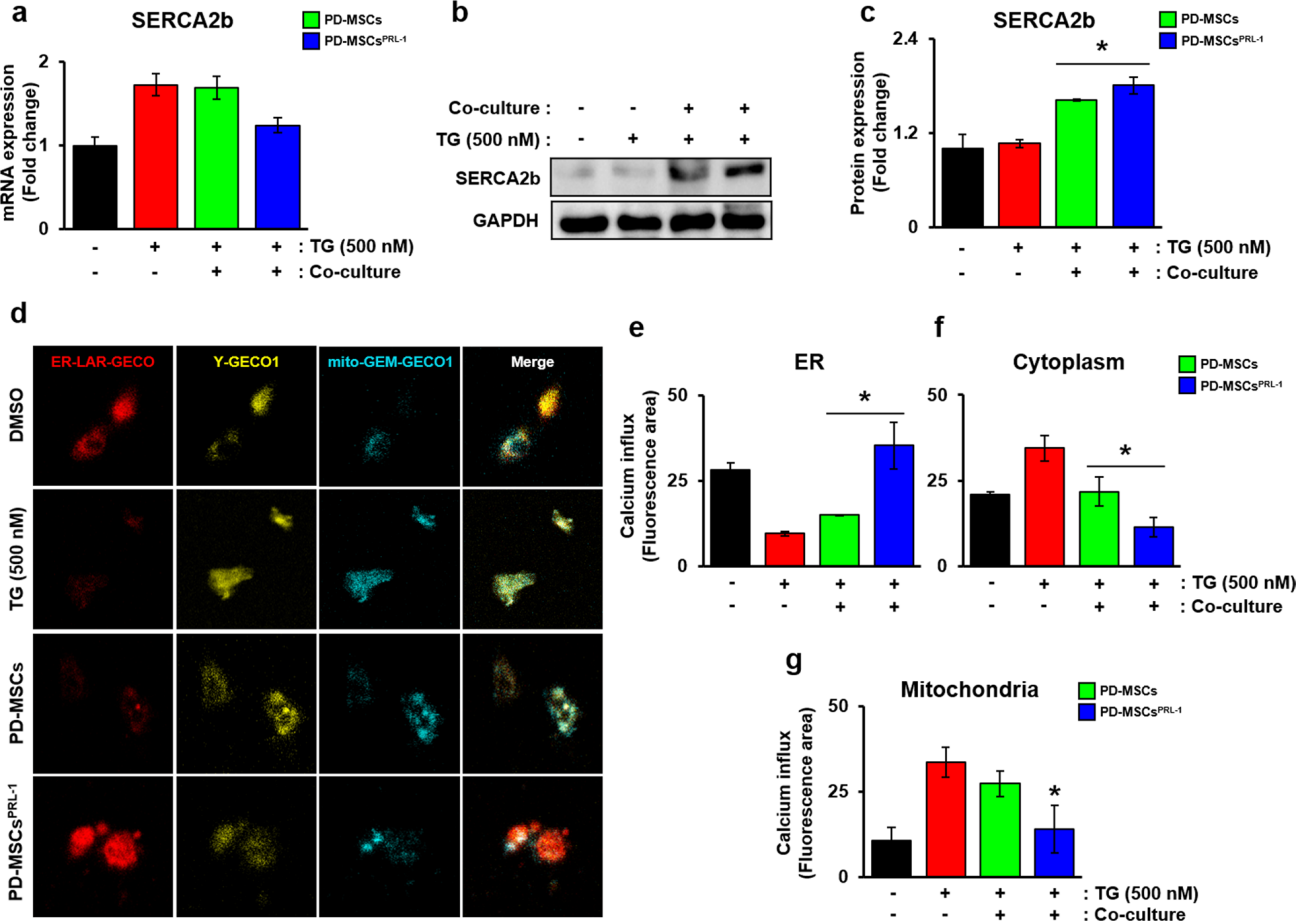
PD-MSCs^PRL−1^ regulated calcium influx in rat hepatocytes. **a** mRNA and **b, c** protein levels of SERCA2b in TG (500 nM)-treated WB-F344 cells cocultured with PD-MSCs or PD-MSCs^PRL−1^ for 24 h. GAPDH was used as a loading control. **d** Representative fluorescence images and **e–g** the fluorescence intensities of calcium influx using ER-LAR GEGO for ER (red), Y-GECO1 for cytoplasm (yellow), and mt-GEM-GECO1 for mitochondria (cyan blue) in TG (500 nM)-treated WB-F344 cells cocultured with PD-MSCs or PD-MSCs^PRL−1^ for 24 h. Data from each group are shown as the means ± SD and were assessed using Student’s t test. *p < 0.05 *vs.* NTx, #*p*< 0.05 *vs.* PD-MSCs. GAPDH, glycraldehyde-3-phosphate dehydrogenase; NTx, nontransplantation; PRL-1, phosphatase of regenerating liver-1; SERCA2b, sarco/endoplasmic reticulum Ca^2+^ ATPase; TG, thapsigargin

To further analyze the concentration of calcium in hepatocytes, we confirmed the mRNA and protein levels of SERCA2b using qRT-PCR and Western blotting, respectively. The mRNA expression levels were increased in the TG-treated group compared to the normal group but decreased in the cocultivation group (Fig. 3a). Although TG treatment reduced the mRNA expression, the protein expression of SERCA2b was significantly increased in cocultured PD-MSCs and PD-MSCs^PRL−1^ (Fig. 3b and c, *p  < 0.05). The mRNA expression of SERCA2b and STIM1, which are calcium sensors in the ER, was significantly increased in PD-MSCs^PRL−1^ (Additional file 1: Fig. S3a and b). To investigate the change in calcium in the ER, cytoplasm, and mitochondria of hepatocytes, we analyzed these organelles using calcium biosensors, including ER-LAR-GECO for the ER, Y-GECO1 for the cytoplasm, and mito-GEM-GECO1 for the mitochondria (Fig. 3d). The influx of ER Ca^2+^ in the TG-treated WB-F344 cells decreased, buts the influx of cytoplasmic and mitochondrial Ca^2+^ was enhanced. Notably, PD-MSC^PRL−1^ cocultivation induced an enhanced ER Ca^2+^ concentration but decreased the influx of cytoplasmic and mitochondrial Ca^2+^ compared to PD-MSCs (Fig. 3e–g, *p  < 0.05). These results indicated that PD-MSC^PRL−1^ cocultivation modulated calcium influx in ER stress-induced hepatocytes.

### PRL-1 regulated EGFR-PI3K-CaM calcium signaling in the BDL-injured rat liver

**Fig. 4:**
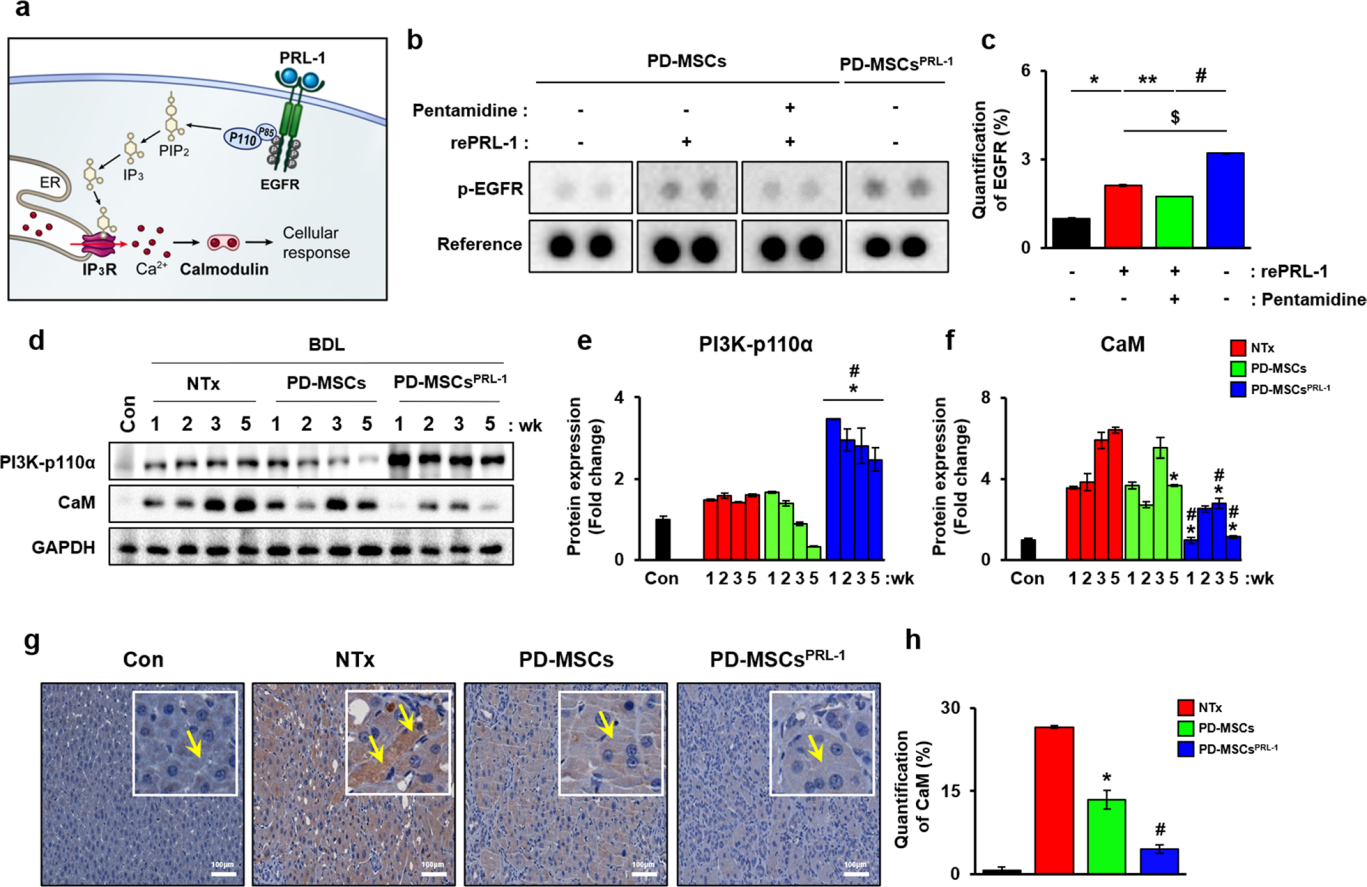
PRL-1 regulated calcium channels by EGFR-PI3K-CaM calcium signaling in BDL-injured rat livers. **a** A schematic diagram showing the effects of PRL-1 via EGFR and downstream factors (e.g., IP3R and CaM). **b** Dot blots and **c** their intensities using phosphorylated RTK assays; *p  < 0.05 *vs.* PD-MSCs, **p  < 0.05 *vs.* PD-MSCs with recombinant PRL-1 (rePRL-1), #*p* < 0.05 *vs.* PD-MSCs with rePRL-1 and pentamidine, $*p* < 0.05 *vs.* PD-MSCs with rePRL-1. **d** Western blotting and **e** and** f** the intensities of PI3K-p110α and CaM in BDL-induced rat livers. GAPDH was used as a loading control. **g** CaM expression and **h** its positive area in BDL-induced rat livers using IHC. Data from each group are shown as the means ± SD and were assessed using Student’s t test. *p  < 0.05 *vs.* NTx, #*p* < 0.05 *vs.* PD-MSCs. BDL, bile duct ligation; CaM, calmodulin; EGFR, epidermal growth factor receptor; GAPDH, glycraldehyde-3-phosphate dehydrogenase; IP3R, inositol trisphosphate receptor; NTx, nontransplantation; PI3K, phosphatidylinositol-3-kinase; PRL-1, phosphatase of regenerating liver-1; RTK, receptor tyrosine kinase

To further investigate PRL-1 calcium signaling, we analyzed the expression of EGFR in hepatocytes injured by pentamidine regardless of cocultivation and recombinant PRL-1 treatment. As shown in Fig. 4a, PRL-1 binds to EGFR and activates PI3K-p110α expression. PIP2 is divided into IP3, and the released IP3 binds to IP3R on the ER membrane. These signaling pathways release Ca^2+^ and regulate the cellular response via the Ca^2+^/CaM complex (Fig. 4a). We performed an RTK dot blot assay and identified which of the 49 different RTKs were phosphorylated (Fig. 4b). Compared to PD-MSCs, PD-MSCs^PRL−1^ strongly activated phosphorylated EGFR after recombinant PRL-1 treatment. Recombinant PRL-1 and pentamidine inhibition treatment reduced the activity of phosphorylated EGFR (Fig. 4c, *p  < 0.05). The protein and mRNA expression of PI3K, which is a downstream factor of EGFR through PRL-1, was increased in the PD-MSC^PRL−1^ group compared to the NTx and PD-MSC groups, but the CaM levels were strongly decreased at 1, 3, and 5 weeks (Fig. 4d–f and Additional file 1: Fig. S4c–e, *p  < 0.05). Endogenous CaM levels in cirrhotic livers in the NTx group were significantly increased, but its expression levels were significantly reduced in the PD-MSC^PRL−1^ group compared to the naïve PD-MSC group (Fig. 4g and h, *p  < 0.05). These results suggest that PRL-1 interacts with EGFR-PI3K-CaM and regulates calcium levels in BDL-injured rat livers.

### Hepatic regenerative effects of PD-MSCs^PRL−1^ in a rat model of BDL

**Fig. 5:**
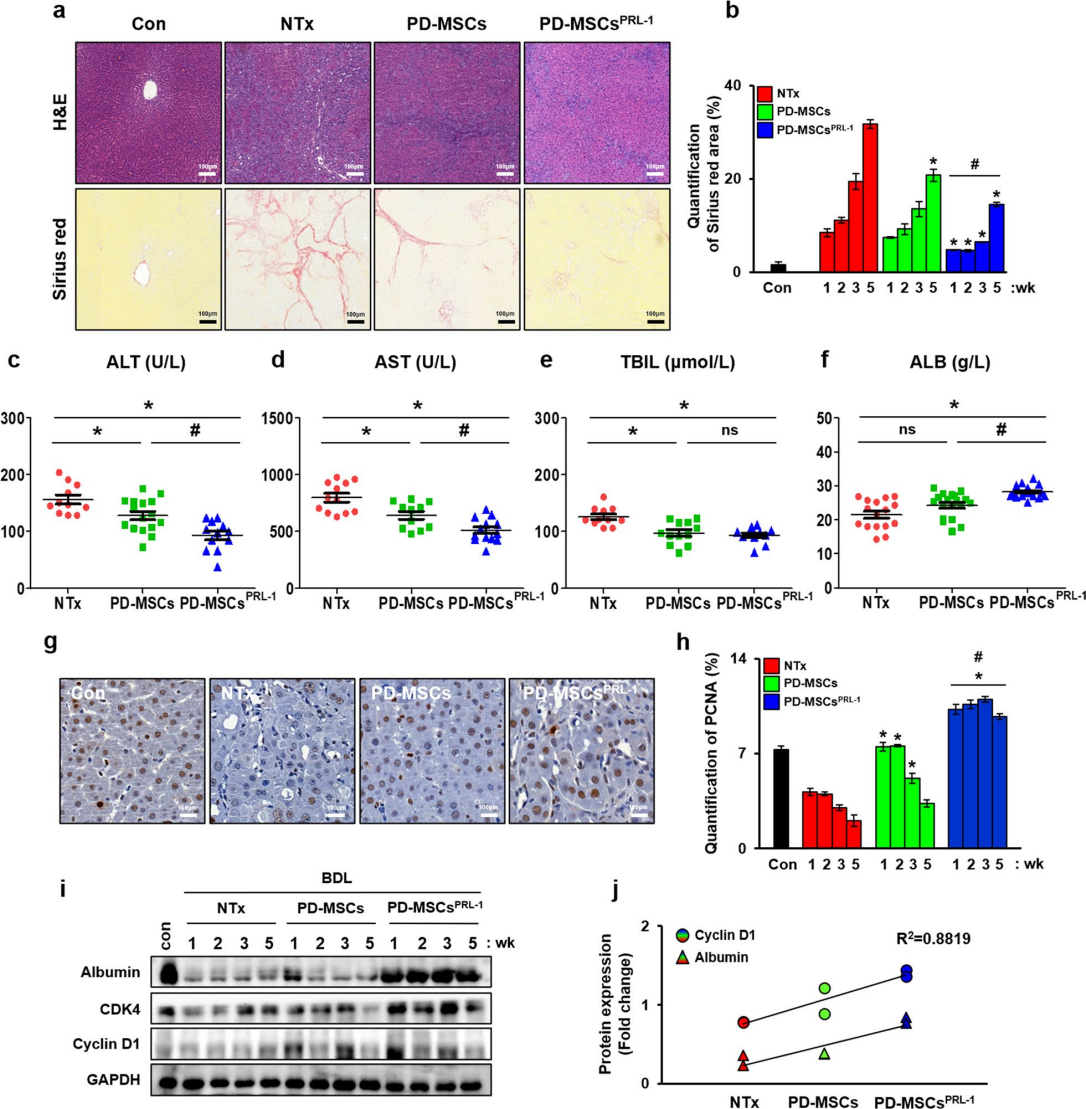
Hepatic regenerative effects of PD-MSCs^PRL−1^ in a rat model of BDL. **a** Representative images of histological analysis of BDL rat liver sections using H&E and Sirius red staining. **b** Quantification of Sirius red positive area. **c-f** Serological analysis of ALT, AST, TBIL, and ALB in individual rat serum samples (*n* = 20/group). **g** PCNA expression and **h** quantification of the PCNA-positive area in a rat liver section from each group determined using IHC. **i** Western blotting of liver regeneration markers (e.g., ALB, CDK4, and cyclin D1) in BDL-induced rat liver protein. GAPDH was used as a loading control. **j** Positive correlation value between ALB and cyclin D1. Data from each group are shown as the means ± SD and were assessed using Student’s t test or one-way ANOVA. *p < 0.05 *vs.* NTx, #*p* < 0.05 *vs.* PD-MSCs. ALT, alanine aminotransferase; ALB, albumin; AST, aspartate aminotransferase; BDL, bile duct ligation; CDK4, cyclin-dependent kinase 4; GAPDH, glycraldehyde-3-phosphate dehydrogenase; NTx, nontransplantation; PCNA, proliferating cell nuclear antigen; PRL-1, phosphatase of regenerating liver-1; TBIL, total bilirubin

**Fig. 6:**
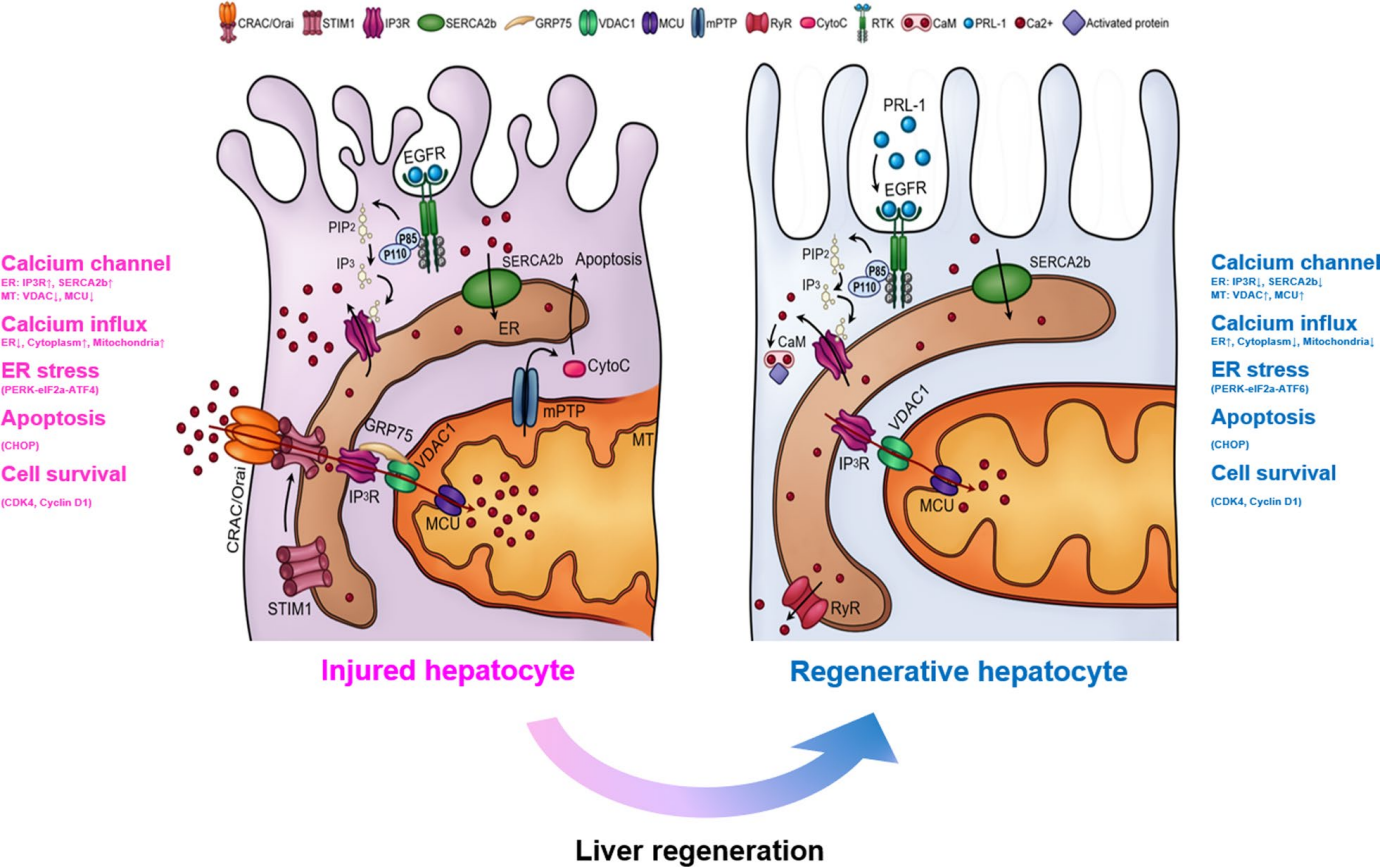
Graphical abstract describing improved hepatic functions and decreased ER stress and calcium influx by PD-MSC^PRL−1^ transplantation in hepatocytes from a rat BDL model via the EGFR-PI3K-CaM signaling pathway. BDL, bile duct ligation; CaM, calmodulin; EGFR, epidermal growth factor receptor; PRL-1, phosphatase of regenerating liver-1; PI3K, phosphatidylinositol-3-kinase

We previously reported that the administration of PD-MSCs^PRL−1^ improved hepatic functions in a rat model of BDL [20]. Collagen accumulation in liver tissues of the NTx group was significantly increased compared to the normal group. However, the number of Sirius red-positive areas significantly decreased in all Tx groups. Notably, antifibrotic effects due to decreased collagen deposition were dramatically observed in the PD-MSC^PRL−1^ group compared to the PD-MSC group (Fig. 5a and b, *p  < 0.05). Blood chemistry analysis revealed that the levels of ALT, AST, and TBIL were decreased in the transplantation groups compared to the NTx group, and the ALB level was significantly increased (Fig. 5c–f, *p  < 0.05). The PD-MSC^PRL−1^ group exhibited substantially decreased ALT and AST and increased ALB levels. To further confirm the proliferation of hepatocytes in the livers of BDL-induced rats following transplantation, we examined proliferating cell nuclear antigen (PCNA) using IHC (Fig. 5g). Compared to naïve PD-MSCs, the positive signal was significantly enhanced in the PD-MSC^PRL−1^ groups (Fig. 5h, *p  < 0.05). The expression of hepatic ALB and proliferation markers (e.g., CDK4 and cyclin D1) in the PD-MSC^PRL−1^ group was remarkably higher than the PD-MSC group (Figs. 5i, 6). The correlation coefficient between ALB and cyclin D1 expression was* R*^2^ = 0.8819 (Fig. 5j). These results suggested that PD-MSCs^PRL−1^ promoted hepatic regeneration in a BDL-injured rat model.

## Discussion

Abnormal calcium levels lead to the ER stress response, which contributes to hepatic lipid accumulation and apoptosis in chronic liver disease [25]. This ER stress response is perturbed due to a calcium depletion-induced accumulation of unfolded/misfolded proteins [26]. The PERK-eIF2α-ATF4 axis mediates the pharmacological ER stress-induced hepatosteatosis that results from lipid homeostasis via several pathways [27]. Therefore, PERK deficiency and increased reactive oxygen species (ROS), due to accumulated lipids and protein aggregation, induce apoptosis in response to ER stress and eIF2α phosphorylation [1]. AMPK inhibition and triglyceride accumulation stimulated hepatic steatosis in mice with liver-specific ATF4 knockout [28]. Increased phosphorylated PERK and CHOP levels also induced intracellular Ca^2+^ overload via the upregulation of Bax expression and increase in the active form of caspase-3 [29]. Therefore, persistent ER stress causes several degenerative diseases, including liver diseases associated with steatosis, via cell death [1]. Many scientists recently reported the efficacies of MSCs on chronic diseases, such as diabetic lung fibrosis, hepatic steatosis and chromium intoxication, by decreasing ER stress [30]. MSCs reduced the ER stress response by decreasing XBP1 and Bip expression via the PERK-Nrf2 signaling pathway [31]. Based on this evidence, our study focused on whether the transplantation of PRL-1-overexpressing PD-MSCs suppressed ER stress-dependent calcium influx in the livers of a rat model of cirrhosis induced by BDL, whether these cells had a positive effect, and elucidation of the mechanisms is involved.

The expression of α-SMA and transforming growth factor 1 and the number of apoptotic and necrotic hepatocytes were decreased in CHOP knockout mice following BDL-induced cirrhosis, which resulted in an ER stress response. Therefore, an increased ER stress response induced the overexpression of CHOP in bile acid-induced hepatocytes via PERK-eIF2α-ATF4 signaling [7]. BM-MSC transplantation in high-fat diet (HFD)-induced rats with NAFLD alleviated ATF4 and CHOP expression [32]. MSC treatment in palmitic acid-induced hepatocytes also reduced CHOP expression, and SERCA2b silencing reversed the ER stress response and calcium homeostasis [33]. These data are similar to our data, which showed that CHOP expression and PERK-eIF2α-ATF4 signaling were increased in the BDL-injured rat model (Fig. 1). We observed changes in calcium channel factors in ER membranes (e.g., SERCA2b and IP3R) and mitochondrial membranes (e.g., VDAC1 and MCU) in BDL-induced rat livers. Following PD-MSC or PD-MSC^PRL−1^ transplantation, the levels of ER stress-related markers and calcium channel markers were dramatically reduced in a rat model of BDL. Treatment of hepatocytes with the SERCA inhibitor TG induced calcium depletion in the ER and the ER stress response via enhancement of the PERK-eIF2α-ATF4-CHOP axis and calcium channel markers in mitochondria. Compared to naïve PD-MSCs, PD-MSCs^PRL−1^ produced dramatic changes in ER Ca^2+^ transport factors in vivo and in vitro (Figs. 1, 2, 3). These results suggest that activated nonparenchymal cells (e.g., Kupffer cells and hepatic stellate cells) and parenchymal cells (e.g., hepatocytes) in a chronic liver disease model were different in the ER stress response and cell death injury [34]. Glucose-regulated protein 78 (GRP78) knockdown alleviated ER stress in activated LX2 cells and restored intracellular calcium levels via the overexpression of SERCA2 [35]. Many reports indicated that treatment of hepatocytes with several bile acids increased the expression of ER stress-related genes (e.g., Bip, XBP1, and CHOP), calcium release and mitochondrial oxidative stress [36]. Bile acid accumulation also activated GRP78 and X-box binding protein 1 (XBP1) [37].

The present study confirmed a significant difference in the expression of PD-MSCs^PRL−1^ compared to naïve PD-MSCs after treatment with 100 μM LCA and cocultured with WB-F344 cells. In contrast to previous results, the expression pattern was similar to after treatment with TG and LCA in WB-F344 cells (Additional file 1: Figures S5, S6). Calcium influx was analyzed in TG-treated hepatocytes using a genetically encoded Ca^2+^ biosensor in the ER, mitochondria, and cytoplasm. PD-MSC^PRL−1^ cocultivation resulted in dynamic changes and recovered calcium levels in the ER (Fig. 3). However, hepatocyte-specific calcium channels in the ER and mitochondria must be further confirmed for isolation from the ER and mitochondria in hepatocytes. Our previous reports demonstrated that the secreted PRL-1 level was significantly higher than naïve cells, function-enhanced PD-MSCs^PRL−1^ were generated [38] and transplantation improved mitochondrial biogenesis and liver function in a BDL-injured rat model. A dramatic increase in the expression of exogenous PRL-1 was confirmed in the transplanted group with PD-MSCs^PRL−1^ and siRNA treated in cocultured rat hepatocytes, and the expression was significantly decreased in PD-MSCs^PRL−1^ compared to naïve PD-MSCs. [20]. PD-MSCs^PRL−1^ promoted antiadipogenic effects via improved IGFBP expression in orbital fibroblasts [39]. Accumulating evidence suggests that excessive intracellular lipids lead to deleterious ER stress and mitochondrial oxidative stress in hepatocytes [40].

Notably, we found that PD-MSCs^PRL−1^ reduced ER stress via PERK-ATF4-CHOP signaling in the BDL-injured rat model and regulated the calcium imbalance between the ER and mitochondria, which resulted in improvements in hepatic function. PERK-dependent eIF2α expression reduced the synthesis of cyclin D1 in stressed cells [41]. To further analyze the PRL-1-induced changes in calcium channels, we performed recombinant PRL-1 treatment and found that it increased IP3R, VDAC1, MCU, and GRP75 expression in LCA-induced hepatocytes (Additional file 1: Figure S7). We also found that PD-MSC^PRL−1^ transplantation improved CDK4 and cyclin D1 expression, which led to the increased proliferation of hepatocytes compared to PD-MSCs (Fig. 5). These data are consistent with Suzuki et al. who reported that PRL-1 inhibited apoptosis with transactivation domains of the p53 transcriptional activator [17]. Following activation of the ER stress response, these molecules localized to the ER in a cell cycle-dependent manner and modulated mitosis [16]. PTPs, including PRL-1, contribute to calcium influx. PTPs are responsible for dephosphorylating EGFR, which indicates a similar specific activity as its Ser/Thr phosphatase activity [42]. Activated EGFR generates Ca^2+^ signals and subsequently controls the Ca^2+^/CaM complex [43]. Based on this evidence, we hypothesized that PRL-1 bound to the first signaling RTKs and affected intracellular calcium levels. Activated EGFR regulates ER stress-dependent calcium signaling, which activates phospholipase C gamma (PLCγ) and the MAPK pathway [44]. We confirmed that phosphorylated EGFR expression was upregulated by recombinant PRL-1 or PD-MSCs^PRL−1^ and activated the expression of the downstream factor PI3K-p110α, but not PI3K-p85, in the PD-MSC^PRL−1^ groups (Fig. 4, Additional file 1: Figure S4). PD-MSC^PRL−1^ transplantation suppressed CaM expression in hepatocytes, which resulted in decreased ER stress and calcium pathway activation. In briefly, we conclude that PRL-1 acts as a cellular trigger in regenerative hepatocytes by binding to the EGFR receptor, which alleviated ER stress and apoptosis and regulated the calcium imbalance between the ER and mitochondria. However, the fundamental mechanism between PRL-1 and EGFR requires further study.

## Conclusion

The administration of PD-MSCs^PRL−1^ decreased ER stress and modulated calcium levels via the EGFR-PI3K-CaM signaling pathway, which led to improved hepatic functions in a rat model of BDL. Therefore, our data on this therapeutic mechanism are useful for the development of next-generation MSC-based stem cell therapy for regenerative medicine in chronic liver disease.

## Supplementary Information

**Additional file 1. Supplementary Fig. 1** Optimal concentration of thapsigargin (TG) in WB-F344. **a** TG was treated with each concentration (100, 500, and 1000 nM) for 6, 12, 24, and 48 h in WB-F344. XBP1 splice form was transformed upon TG 24 h treatment. GAPDH was used as loading control. GAPDH, glycraldehyde-3-phosphate dehydrogenase; TG, tapsigargin. **Supplementary Fig. 2** PD-MSCs^PRL-1^ regulated calcium channels in a rat BDL and hepatocyte treated with TG. **a**–**d** mRNA levels of calcium channels (e.g., IP3R, GRP75, VDAC1, and MCU) in a rat model with BDL. **e**-**i** mRNA levels of calcium channels (e.g., IP3R, GRP75, VDAC1, MCU, and CaM) exposed to TG (500 nM) for 24 h in WB-F344 by qRT-PCR. Data from each group are shown as the means ± SD and were assessed using Student’s t-test. *p < 0.05 vs. NTx, #p < 0.05 vs. PD-MSCs. BDL, bile duct ligation; CaM, calmodulin; GRP75, glucose regulated protein 75; IP3R, inositol trisphosphate receptor; MCU, mitochondria calcium uniporter; NTx, nontransplantation; PRL-1, phosphatase of regenerating liver-1; TG, thapsigargin; VDAC1, voltage dependent anion channel 1. **Supplementary Fig. 3** Increased expression of SERCA2b and STIM1 in PD-MSCs^PRL-1^ groups in a rat model with BDL. **a**, **b** mRNA levels of ER-specific Ca^2+^ channel factor SERCA2b and Ca^2+^ sensor factor STIM1 in a rat model with BDL. Data from each group are shown as the means ± SD and were assessed using Student’s t-test. *p < 0.05 vs. NTx, #p < 0.05 vs. PD-MSCs. BDL, bile duct ligation; NTx, nontransplantation; SERCA2b, sarco/endoplasmic reticulum Ca^2+^ ATPase; PRL-1, phosphatase of regenerating liver-1; STIM1, stromal interaction molecule 1. **Supplementary Fig. 4** PRL-1 regulates EGFR-PI3K-CaM calcium signaling in a BDL-injured rat liver and rat hepatocyte treated LCA. **a** mRNA levels of CaM and **b** PI3K-p85 in a rat model with BDL. **c**-**e** mRNA levels of intracellular calcium signaling (e.g., EGFR, PI3K, and CaM) induced LCA (100 μM) and treated recombinant PRL-1 (rePRL-1; 500 pg) in WB-F344. Data from each group are shown as the means ± SD and were assessed using Student’s t-test. *p < 0.05 vs. NTx, #p < 0.05 vs. PD-MSCs. BDL, bile duct ligation; CaM, calmodulin; EGFR, epidermal growth factor receptor; NTx, nontransplantation; PI3K, phosphatidylinositol-3-kinase. **Supplementary Fig. 5** PD-MSCs^PRL-1^ modulated calcium channels in a rat hepatocyte treated with LCA by qRT-PCR. **a-g** mRNA levels related to intracellular calcium transport channels factors (e.g., SERCA2b, IP3R, VDAC1, MCU, GRP75, CaM, and STIM1) induced LCA (100 μM) and co-cultivation with naïve PD-MSCs or PD-MSCs^PRL-1^ in WB-F344. Data from each group are shown as the means ± SD and were assessed using Student’s t-test. *p < 0.05 vs. LCA (100 μM), #p < 0.05 vs. PD-MSCs. CaM, calmodulin; GRP75, glucose regulated protein 75; IP3R, inositol trisphosphate receptor; LCA, lithocholic acid; MCU, mitochondria calcium uniporter; PRL-1, phosphatase of regenerating liver-1; SERCA2b, sarco/endoplasmic reticulum Ca^2+^ ATPase; STIM1, stromal interaction molecule 1; VDAC1, voltage dependent anion channel 1. **Supplementary Fig. 6** PD-MSCs^PRL-1^ regulated calcium channels in a rat hepatocyte treated with LCA and TG by western blotting. **a** Protein expression and **b-f** their intensities of intracellular calcium transport channels factors (e.g., SERCA2b, IP3R, VDAC1, MCU, and GRP75) induced LCA (100 μM) and co-cultivation with naïve PD-MSCs or PD-MSCs^PRL-1^ in WB-F344. **g** Protein expression and h-j their intensities of intracellular calcium transport channels factors (e.g., SERCA2b, and MCU) and liver regeneration factor induced LCA (100 μM) and co-cultivation with naïve PD-MSCs or PD-MSCs^PRL-1^ and Pentamidine (10 μg) treated in rat primary hepatocytes. GAPDH and α-tubulin was used as loading control. Data from each group are shown as the means ± SD and assessed using Student’s t-test. *p < 0.05 vs. LCA (100 μM), #p < 0.05 vs. PD-MSCs, $p < 0.05 vs. PD-MSCs or PD-MSCs^PRL-1^. ALB, Albumin; GAPDH, glycraldehyde-3-phosphate dehydrogenase; GRP75, glucose regulated protein 75; IP3R, inositol trisphosphate receptor; LCA, lithocholic acid; MCU, mitochondria calcium uniporter; PRL-1, phosphatase of regenerating liver-1; SERCA2b, sarco/endoplasmic reticulum Ca^2+^ ATPase; TG, Thapsigargin; VDAC1, voltage dependent anion channel 1. **Supplementary Fig. 7** Recombinant PRL-1 increased calcium channels in a rat hepatocyte treated with LCA. **a-g** mRNA levels of intracellular calcium transport channels factors (e.g., SERCA2b, IP3R, VDAC1, MCU, GRP75, CaM, and STIM1) induced LCA (100 μM) and treated recombinant PRL-1 (rePRL-1; 500 pg) in WB-F344. Data from each group are shown as the means ± SD and assessed using Student’s t-test. *p < 0.05 vs. LCA (100 μM). CaM, calmodulin; GRP75, glucose regulated protein 75; IP3R, inositol trisphosphate receptor; LCA, lithocholic acid; MCU, mitochondria calcium uniporter; PRL-1, phosphatase of regenerating liver-1; SERCA2b, sarco/endoplasmic reticulum Ca^2+^ ATPase; VDAC1, voltage dependent anion channel 1.

## Data Availability

The data that support the findings of this study are available from the corresponding author upon reasonable request.

## Abbreviations

ALT: Alanine aminotransferase
ALB: Albumin
α-MEM: Alpha-modified minimal essential medium
AST: Aspartate aminotransferase
ATF4: Activating transcription factor 4
BDL: Bile duct ligation
CaM: Calmodulin
CDK4: Cyclin-dependent kinase 4
CHOP: C/EBP homologous protein
DAPI: 4′,6-Diamidino-2-phenylindole
EGFR: Epidermal growth factor receptor
FBS: Fetal bovine serum
GAPDH: Glyceraldehyde 3-phosphate dehydrogenase
GFP: Green fluorescent protein
GRP75: Glucose regulated protein 75
GRP78: Glucose regulated protein 78
HFD: High-fat diet
hFGF-4: Human fibroblast growth factor-4
H&E: Hematoxylin & eosin
IP3R: Inositol trisphosphate receptor
LCA: Lithocholic acid
MCU: Mitochondria calcium uniporter
NBF: Neutral buffered formalin
NTx: Nontransplantation
PERK: PKR-like ER kinase
PCNA: Proliferating cell nuclear antigen
PD-MSCs: Placenta-derived mesenchymal stem cells
p-eIF2α: Phosphorylated eIF2 alpha
PI3K: Phosphatidylinositol-3-kinase
PRL-1: Phosphatase of regenerating liver-1
P/S: Penicillin/streptomycin
RTK: Receptor tyrosine kinases
SERCA2b: Sarco/endoplasmic reticulum Ca^2^^+^ ATPase
SD: Sprague–Dawley
STIM1: Stromal interaction molecule 1
TBIL: Total bilirubin
TG: Thapsigargin
VDAC1: Voltage-dependent anion channel 1
XBP-1: X-box binding protein 1
