# Supplementary material for: Activation of the EGFR-PI3K-CaM pathway by PRL-1-overexpressing placenta-derived mesenchymal stem cells ameliorates liver cirrhosis via ER stress-dependent calcium
Source: Stem Cell Res Ther. 2021 Oct 24;12:551. doi: 10.1186/s13287-021-02616-y (PMC8543968; doi:10.1186/s13287-021-02616-y)
Supplement: Supplementary file 1 — Additional file 1. Supplementary Fig. 1 Optimal concentration of thapsigargin (TG) in WB-F344. a TG was treated with each concentration (100, 500, and 1000 nM) for 6, 12, 24, and 48 h in WB-F344. XBP1 splice form was transformed upon TG 24 h treatment. GAPDH was used as loading control. GAPDH, glycraldehyde-3-phosphate dehydrogenase; TG, tapsigargin. Supplementary Fig. 2 PD-MSCsPRL-1 regulated calcium channels in a rat BDL and hepatocyte treated with TG. a–d mRNA levels of calcium channels (e.g., IP3R, GRP75, VDAC1, and MCU) in a rat model with BDL. e-i mRNA levels of calcium channels (e.g., IP3R, GRP75, VDAC1, MCU, and CaM) exposed to TG (500 nM) for 24 h in WB-F344 by qRT-PCR. Data from each group are shown as the means ± SD and were assessed using Student’s t-test. *p < 0.05 vs. NTx, #p < 0.05 vs. PD-MSCs. BDL, bile duct ligation; CaM, calmodulin; GRP75, glucose regulated protein 75; IP3R, inositol trisphosphate receptor; MCU, mitochondria calcium uniporter; NTx, nontransplantation; PRL-1, phosphatase of regenerating liver-1; TG, thapsigargin; VDAC1, voltage dependent anion channel 1. Supplementary Fig. 3 Increased expression of SERCA2b and STIM1 in PD-MSCsPRL-1 groups in a rat model with BDL. a, b mRNA levels of ER-specific Ca2+ channel factor SERCA2b and Ca2+ sensor factor STIM1 in a rat model with BDL. Data from each group are shown as the means ± SD and were assessed using Student’s t-test. *p < 0.05 vs. NTx, #p < 0.05 vs. PD-MSCs. BDL, bile duct ligation; NTx, nontransplantation; SERCA2b, sarco/endoplasmic reticulum Ca2+ ATPase; PRL-1, phosphatase of regenerating liver-1; STIM1, stromal interaction molecule 1. Supplementary Fig. 4 PRL-1 regulates EGFR-PI3K-CaM calcium signaling in a BDL-injured rat liver and rat hepatocyte treated LCA. a mRNA levels of CaM and b PI3K-p85 in a rat model with BDL. c-e mRNA levels of intracellular calcium signaling (e.g., EGFR, PI3K, and CaM) induced LCA (100 μM) and treated recombinant PRL-1 (rePRL-1; 500 pg) in WB-F344. Data [file 13287_2021_2616_MOESM1_ESM.pdf]

Supplementary Fig. 1 SH Kim et al.

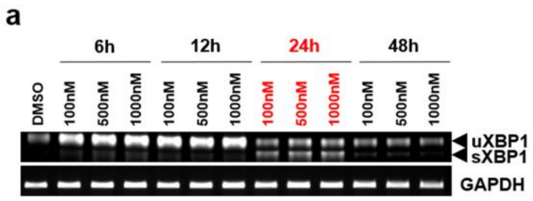

Supplementary Fig. 1 Optimal concentration of thapsigargin (TG) in WB-F344. **a** TG was treated with each concentration (100, 500, and 1000 nM) for 6, 12, 24, and 48 h in WB-F344. XBP1 splice form was transformed upon TG 24 h treatment. GAPDH was used as loading control. GAPDH, glycraldehyde-3-phosphate dehydrogenase; TG, tapsigargin

Supplementary Fig. 2 **SH Kim et al.**

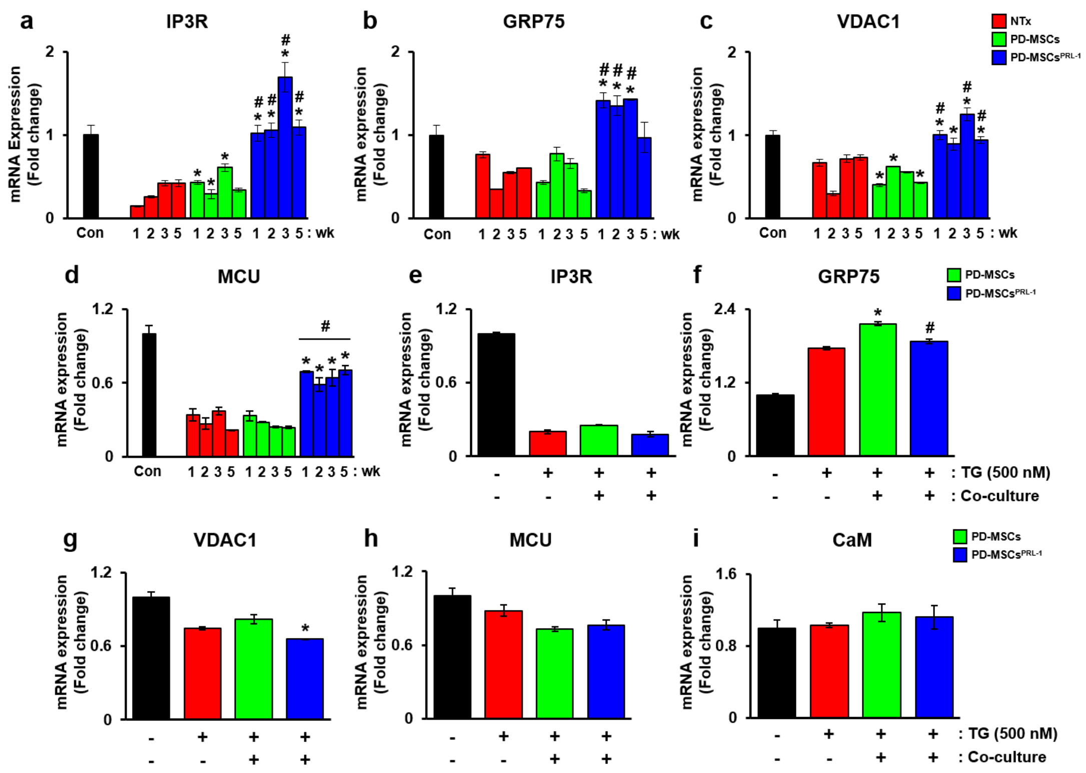

Supplementary Fig. 2 PD-MSCs^{PRL-1} regulated calcium channels in a rat BDL and hepatocyte treated with TG. **a-d** mRNA levels of calcium channels (e.g., IP3R, GRP75, VDAC1, and MCU) in a rat model with BDL. **e-i** mRNA levels of calcium channels (e.g., IP3R, GRP75, VDAC1, MCU, and CaM) exposed to TG (500 nM) for 24 h in WB-F344 by qRT-PCR. Data from each group are shown as the means ± SD and were assessed using Student's t-test. *p < 0.05 vs. NTx, #p < 0.05 vs. PD-MSCs. BDL, bile duct ligation; CaM, calmodulin; GRP75, glucose regulated protein 75; IP3R, inositol trisphosphate receptor; MCU, mitochondria calcium uniporter; NTx, nontransplantation; PRL-1, phosphatase of regenerating liver-1; TG, thapsigargin; VDAC1, voltage dependent anion channel 1

Supplementary Fig. 3 **SH Kim et al.**

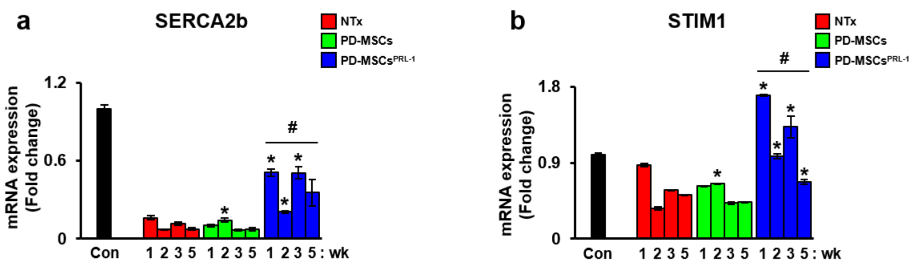

Supplementary Fig. 3 Increased expression of SERCA2b and STIM1 in PD-MSCs^{PRL-1} groups in a rat model with BDL. **a**, **b** mRNA levels of ER-specific Ca^{2+} channel factor SERCA2b and Ca^{2+} sensor factor STIM1 in a rat model with BDL. Data from each group are shown as the means ± SD and were assessed using Student’s t-test. *p < 0.05 vs. NTx, #p < 0.05 vs. PD-MSCs. BDL, bile duct ligation; NTx, nontransplantation; SERCA2b, sarco/endoplasmic reticulum Ca^{2+} ATPase; PRL-1, phosphatase of regenerating liver-1; STIM1, stromal interaction molecule 1

Supplementary Fig. 4 **SH Kim et al.**

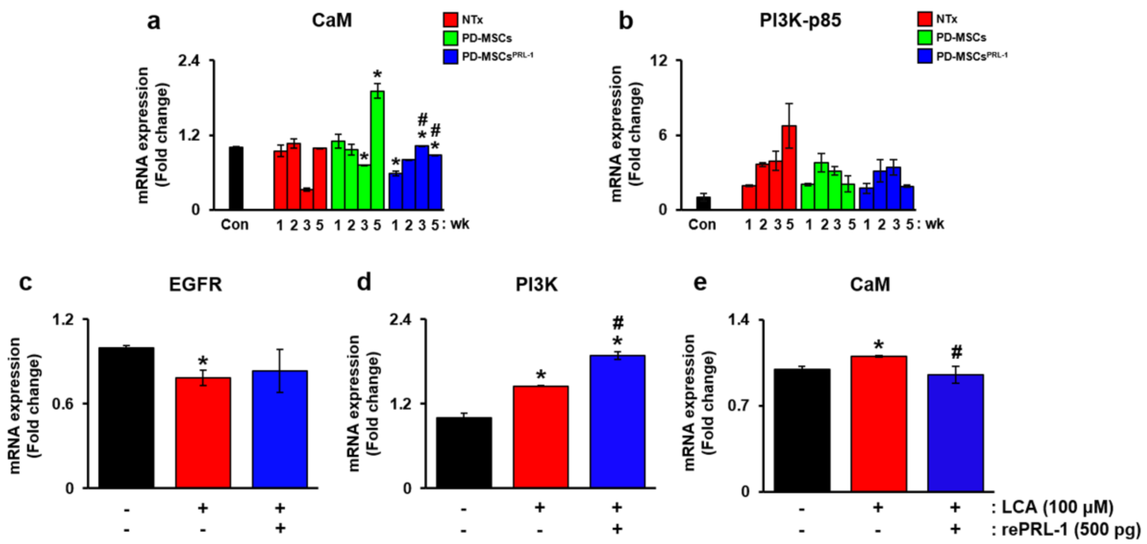

Supplementary Fig. 4 PRL-1 regulates EGFR-PI3K-CaM calcium signaling in a BDL-injured rat liver and rat hepatocyte treated LCA. **a** mRNA levels of CaM and **b** PI3K-p85 in a rat model with BDL. **c-e** mRNA levels of intracellular calcium signaling (e.g., EGFR, PI3K, and CaM) induced LCA (100 µM) and treated recombinant PRL-1 (rePRL-1; 500 pg) in WB-F344. Data from each group are shown as the means ± SD and were assessed using Student's t-test. *$p < 0.05$ vs. NTx, #$p < 0.05$ vs. PD-MSCs. BDL, bile duct ligation; CaM, calmodulin; EGFR, epidermal growth factor receptor; NTx, nontransplantation; PI3K, phosphatidylinositol-3-kinase

Supplementary Fig. 5 **SH Kim et al.**

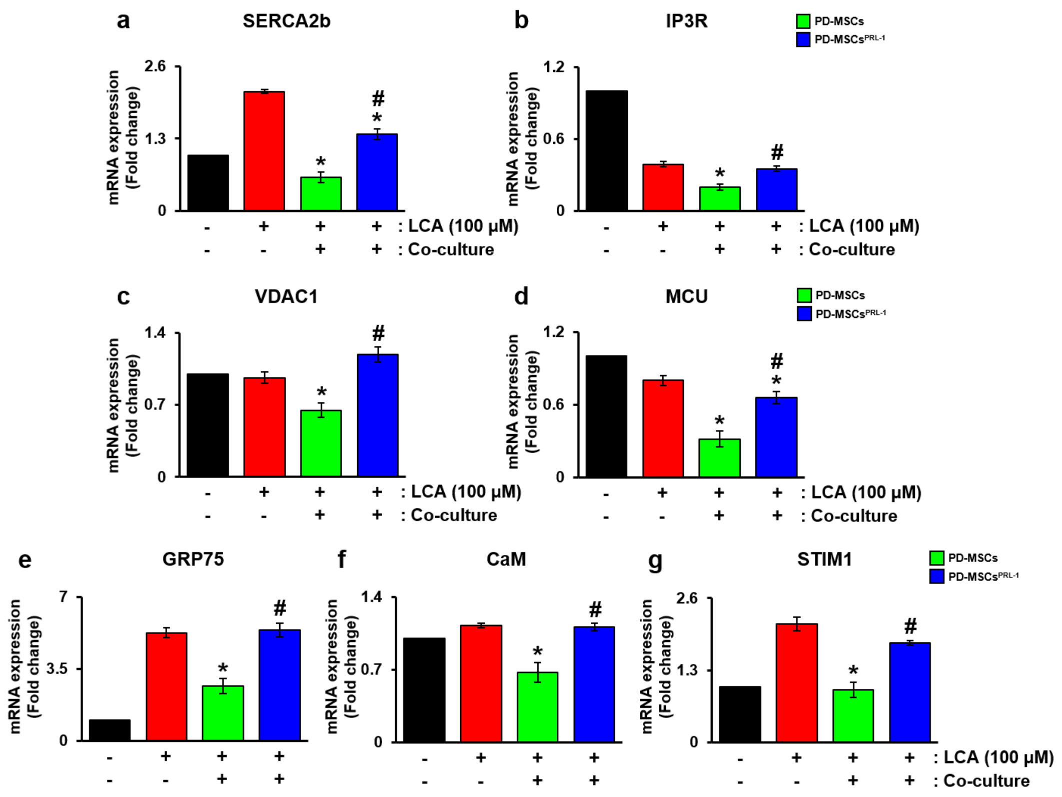

Supplementary Fig. 5 PD-MSCs^{PRL-1} modulated calcium channels in a rat hepatocyte treated with LCA by qRT-PCR. **a-g** mRNA levels related to intracellular calcium transport channels factors (e.g., SERCA2b, IP3R, VDAC1, MCU, GRP75, CaM, and STIM1) induced LCA (100 μM) and co-cultivation with naïve PD-MSCs or PD-MSCs^{PRL-1} in WB-F344. Data from each group are shown as the means ± SD and were assessed using Student's t-test. *p < 0.05 vs. LCA (100 μM), #p < 0.05 vs. PD-MSCs. CaM, calmodulin; GRP75, glucose regulated protein 75; IP3R, inositol trisphosphate receptor; LCA, lithocholic acid; MCU, mitochondria calcium uniporter; PRL-1, phosphatase of regenerating liver-1; SERCA2b, sarco/endoplasmic reticulum Ca^{2+} ATPase; STIM1, stromal interaction molecule 1; VDAC1, voltage dependent anion channel 1

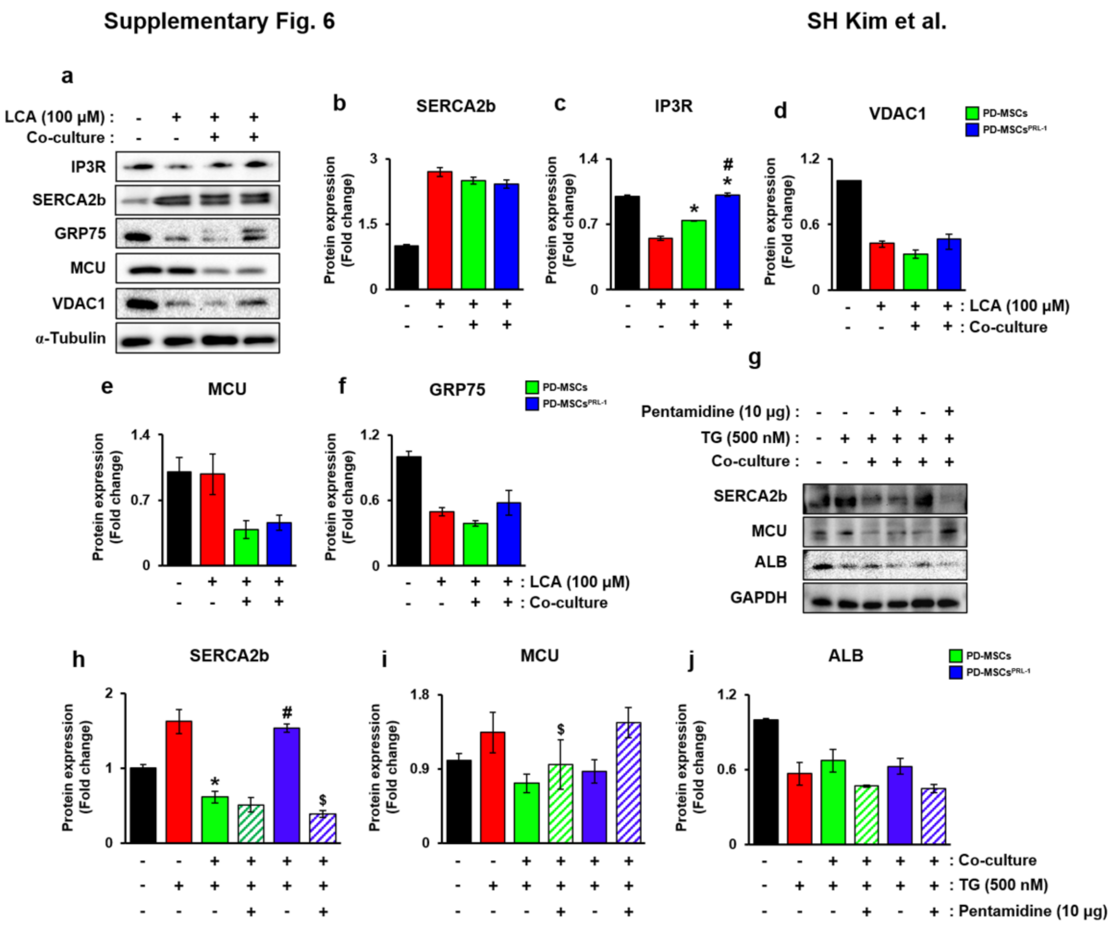

Supplementary Fig. 6 PD-MSCs$^{PRL\text{-}1}$ regulated calcium channels in a rat hepatocyte treated with LCA and TG by western blotting. **a** Protein expression and **b-f** their intensities of intracellular calcium transport channels factors (e.g., SERCA2b, IP3R, VDAC1, MCU, and GRP75) induced LCA (100 μM) and co-cultivation with naïve PD-MSCs or PD-MSCs$^{PRL\text{-}1}$ in WB-F344. **g** Protein expression and **h-j** their intensities of intracellular calcium transport channels factors (e.g., SERCA2b, and MCU) and liver regeneration factor induced LCA (100 μM) and co-cultivation with naïve PD-MSCs or PD-MSCs$^{PRL\text{-}1}$ and Pentamidine (10 μg) treated in rat primary hepatocytes. GAPDH and α-tubulin was used as loading control. Data from each group are shown as the means ± SD and assessed using Student's t-test. *$p < 0.05$ vs. LCA (100 μM), #$p < 0.05$ vs. PD-MSCs, \$$p < 0.05$ vs. PD-MSCs or PD-MSCs$^{PRL\text{-}1}$. ALB, Albumin; GAPDH, glycraldehyde-3-phosphate dehydrogenase; GRP75, glucose regulated protein 75; IP3R, inositol trisphosphate receptor; LCA, lithocholic acid; MCU, mitochondria calcium uniporter; PRL-1, phosphatase of regenerating liver-1; SERCA2b,

sarco/endoplasmic reticulum Ca^{2+} ATPase; TG, Thapsigargin; VDAC1, voltage dependent anion channel 1

Supplementary Fig. 7 **SH Kim et al.**

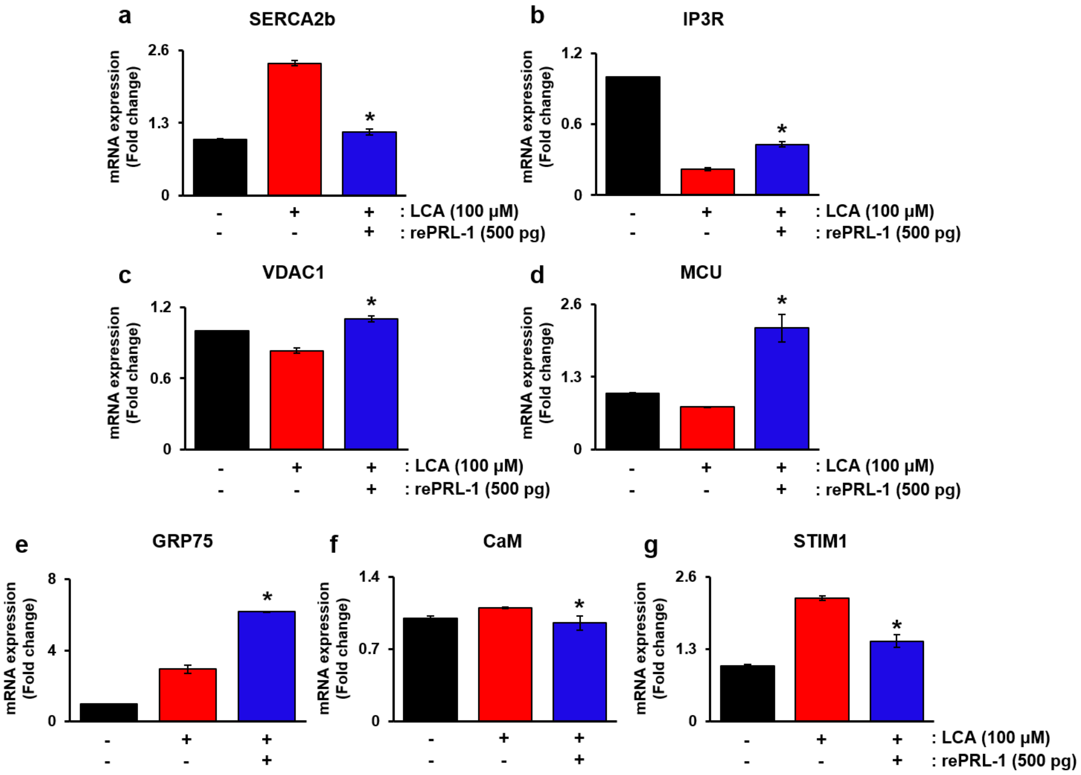

Supplementary Fig. 7 Recombinant PRL-1 increased calcium channels in a rat hepatocyte treated with LCA. **a-g** mRNA levels of intracellular calcium transport channels factors (e.g., SERCA2b, IP3R, VDAC1, MCU, GRP75, CaM, and STIM1) induced LCA (100 μM) and treated recombinant PRL-1 (rePRL-1; 500 pg) in WB-F344. Data from each group are shown as the means ± SD and assessed using Student's t-test. *p < 0.05 vs. LCA (100 μM). CaM, calmodulin; GRP75, glucose regulated protein 75; IP3R, inositol trisphosphate receptor; LCA, lithocholic acid; MCU, mitochondria calcium uniporter; PRL-1, phosphatase of regenerating liver-1; SERCA2b, sarco/endoplasmic reticulum Ca^{2+} ATPase; VDAC1, voltage dependent anion channel 1
